# Who Uses Glucosamine and Why? A Study of 266,848 Australians Aged 45 Years and Older

**DOI:** 10.1371/journal.pone.0041540

**Published:** 2012-07-30

**Authors:** David Sibbritt, Jon Adams, Chi-Wai Lui, Alex Broom, Jonathan Wardle

**Affiliations:** 1 School of Medicine and Public Health, University of Newcastle, Callaghan, New South Wales, Australia; 2 Faculty of Nursing Midwifery and Health, University of Technology Sydney, Sydney, New South Wales, Australia; 3 School of Population Health, University of Queensland, Brisbane, Queensland, Australia; 4 School of Social Science, University of Queensland, Brisbane, Queensland, Australia; 5 Faculty of Nursing Midwifery and Health, University of Technology Sydney, Sydney, New South Wales, Australia; The University of Texas M. D. Anderson Cancer Center, United States of America

## Abstract

**Objectives:**

There has been a dramatic increase in the use of complementary medicines over recent decades. Glucosamine is one of the most commonly used complementary medicines in Western societies. An understanding of glucosamine consumption is of significance for public health and future health promotion. This paper, drawing upon the largest dataset to date with regards to glucosamine use (n = 266,844), examines the use and users of glucosamine amongst a sample of older Australians.

**Design:**

Analysis of the self-reported data on use of glucosamine, demographics and health status as extracted from the dataset of the 45 and Up Study, which is the largest study of healthy ageing ever undertaken in the Southern Hemisphere involving over 265,000 participants aged 45 and over.

**Results:**

Analysis reveals that 58,630 (22.0%) participants reported using glucosamine in the 4 weeks prior to the survey. Use was higher for those who were female, non-smokers, residing in inner/outer regional areas, with higher income and private health insurance. Of all the health conditions examined only osteoarthritis was positively associated with use of glucosamine, while cancer, heart attack or angina and other heart disease were all negatively associated with glucosamine use.

**Conclusions:**

This study suggests that a considerable proportion of the Australia population aged 45 and over consume glucosamine. There is a need for health care practitioners to enquire with their patients about their use of glucosamine and for further attention to be directed to providing good quality information for patients and providers with regards to glucosamine products.

## Introduction

The use of dietary supplements and complementary and alternative medicine (CAM) has grown rapidly over the past few decades [1], [2] – a growth which has been associated with lifestyle changes, population ageing, as well as the increasing prevalence of chronic diseases (often proving difficult for conventional medicine to address) [3]–[5].

Glucosamine is one of the most commonly used CAM with recent research identifying 20% of the US adult population using glucosamine [6]. In Australia, glucosamine is the most commonly recommended CAM by GPs and community pharmacists, with 85.2% of GPs and 94.7% of Australian community pharmacists recommending glucosamine to patients [7], [8]. Three recent studies on the use of conventional and complementary medicines among Australians reported a glucosamine use of 15% (respondents aged over 50) [9], 33% (respondents aged 60 or over) [10], and 24% (an average respondent age of 66) [11].

Glucosamine is commonly used as a preventive measure and treatment for joint problems and osteoarthritis [12], [13]. For example, a US study of 612 patients with osteoarthritis, rheumatoid arthritis, or fibromyalgia found that glucosamine was the most commonly used CAM (27% of patients reporting use) [14]. Yet the evidence base for the effectiveness of glucosamine as a treatment for osteoarthritis remains inconclusive and controversial [15]. Meta-analyses of the effectiveness of glucosamine in preventing or ameliorating the symptoms of osteoarthritis have produced mixed findings [16]–[21]. Moreover, it has been suggested that this heterogeneity of results could be exacerbated by factors such as varying formulations (i.e. glucosamine sulfate vs. hydrochloride), variations in quality, industry bias, and/or methodological variability [22], [23].

Despite the extensive use of glucosamine, the topic has received little attention to date. While previous broad CAM-focused studies have provided prevalence rates for glucosamine use [6], there has been little focused examination of the profile of glucosamine users and the various factors influencing use. To date, only one study has examined the prevalence and factors associated with glucosamine use among a general population [24] showing glucosamine use among a random sample of 7,652 Canadians over a 5-year period (1996–7 to 2001–2) increased substantially, from 0.9% to 4.7% for men and from 1.3% to 8.2% for women. This Canadian study found glucosamine use was higher among women and increased with age and suggested glucosamine may be used for the management of arthritis symptoms and/or as a preventive health care measure. Glucosamine use was also found to be associated with geographical region (higher use in western Canada), arthritis, back/neck pain, higher calcium intake, and regular physical activity [24].

The adverse effects of glucosamine have been well studied and are considered uncommon and minor [25]–[27]. Nevertheless, there are reports suggesting an interaction between glucosamine and warfarin [28], [29] − a concern given the common usage of warfarin (the most widely used oral anticoagulant in Australia) [30] and the high rates of patient non-disclosure of CAM use to conventional practitioners [31].

Given the rise of population ageing [32], increasing consumer interest in the value of healthy eating, exercise and dietary supplements [3], and growth in public awareness of the importance of preventive health [33], an empirical analysis of the prevalence and pattern of glucosamine consumption is of significance for public health, future health promotion and the regulation of consumer CAM products.

An understanding of the prevalence and pattern of glucosamine use is crucial for an assessment of its benefits as well as the risk of potential interactions with drugs such as warfarin in the general population [34]. In response, this paper reports the findings of a comprehensive study that examines the use and users of glucosamine amongst a large sample of Australians (n = 266,848) aged 45 years and older.

## Method

### Sample

This research utilised data collected through the 45 and Up Study, which is the largest study of healthy ageing conducted in the Southern Hemisphere and analyses data from over 266,000 men and women aged 45 and older who reside in the State of New South Wales, Australia. The study is described in detail elsewhere [35], but briefly participants for this study were randomly selected from the Medicare Australia database, which provides virtually complete coverage of the general population. Participants entered the study by completing a baseline postal questionnaire and providing written consent to have their health followed over time.

The 45 and Up study has the approval of the University of New South Wales Ethics Committee and informed written consent of the study participants was obtained. Participants joined the study by completing a postal questionnaire. Recruitment began in February 2006 and these analyses relate to the 266,848 participants joining the study to the end of December, 2009. The overall response rate to the baseline questionnaire is estimated to be 17.9%. The 45 and Up study sample has excellent heterogeneity and is reasonably representative of the (State of) New South Wales population; has a response rate comparable to similar studies internationally and in Australia; and is among the most representative large scale cohort studies in the world [36].

### Use of Glucosamine

Participants were defined as being a glucosamine user if they answered ‘yes’ to the following question: ‘In the past 4 weeks have you taken glucosamine.’

### Demographic Measures

Area of residence was assigned according to the Accessibility Remoteness Index of Australia Plus [37] score for each participant’s postcode. Participants were asked about their current marital status, highest educational qualification they had completed, annual household income, and their level of healthcare insurance.

### Health Status Measures

Participants were asked to rate their overall health and overall quality of life on a five-point Likert scale. They were also asked about their history of smoking and amount of alcohol consumption. Participants were provided with a list of diseases (e.g. osteoarthritis, osteoporosis, asthma, cancer) and asked if they had been treated for any of these diseases in the last month. Answers to this question were used to determine whether a participant had a particular disease or not.

### Statistical Analyses

The demographic and health status characteristics of glucosamine users and non-users were compared using chi-square tests. Logistic regression modelling, that included all demographic and health status characteristics variables, was conducted using a backward stepwise method, to parsimoniously predict use of glucosamine. The model building process utilised the likelihood ratio test to compare competing models. In response to the large sample size and multiple comparisons, a p-value <0.005 was adopted for statistical significance. All analyses were conducted using the statistical software program SAS, version 9.2 (SAS Institute, Inc., Cary, NC, 2008).

## Results

There were 266,844 participants who answered the question regarding consumption of glucosamine, of which 58,630 (22.0%) indicated that they had taken glucosamine in the 4 weeks prior to the survey.

A comparison between participants who used glucosamine and those who did not use glucosamine by demographic characteristics is provided in Table 1. Use of glucosamine is highest among females and those aged 60–79 years. Use of glucosamine was higher for those participants: residing in inner regional areas; having a trade, certificate or diploma education; having an annual household income of $20000–$69999; being widowed, divorced or separated; and having private health insurance (p<0.0001 for all variables).

**Table 1:** Demographic characteristic of people aged 45 years and older by glucosamine use.

		Use of Glucosamine			
Demographic Characteristics*		Yes		No	
		(n = 58,630)		(n = 208,214)	
		%	(95% C.I.)	%	(95% C.I.)
**Sex**	Female	60.5	(60.1, 60.9)	51.7	(51.5, 51.9)
	Male	39.5	(39.1, 39.9)	48.3	(48.1, 48.5)
**Age (years)**	45–49	7.6	(7.4, 7.8)	14.6	(14.4, 14.8)
	50–59	30.9	(30.5, 31.3)	33.8	(33.6, 34.0)
	60–69	33.0	(32.6, 33.4)	26.3	(26.1, 26.5)
	70–79	18.6	(18.3, 18.9)	15.0	(14.8, 15.2)
	80+	9.9	(9.7, 10.1)	10.3	(10.2, 10.4)
**Place of**	Major city	44.1	(43.7, 44.5)	45.1	(44.9, 45.3)
**Residence**	Inner regional	36.4	(36.0, 36.8)	35.1	(34.9, 35.3)
	Outer regional	17.9	(17.6, 18.2)	17.7	(17.5, 17.9)
	Remote/veryremote	1.6	(1.5, 1.7)	2.1	(2.0, 2.2)
**Education**	School Certificateor less	33.6	(33.2, 34.0)	34.5	(34.3, 34.7)
	Higher School Certificate	9.8	(9.6, 10.0)	10.0	(9.9, 10.1)
	Trade/certificate/diploma	33.2	(32.8, 33.6)	32.1	(31.9, 32.3)
	Tertiary	23.4	(23.1, 23.7)	23.4	(23.2, 23.6)
**Annual**	< $20000	24.0	(23.7, 24.3)	25.5	(25.3, 25.7)
**Household**	$20000–$49999	34.3	(33.9, 34.7)	30.8	(30.6, 31.0)
**Income**	$50000–$69999	13.6	(13.3, 13.9)	13.2	(13.1, 13.3)
	≥ $70000	28.1	(27.7, 28.5)	30.5	(30.3, 30.7)
**Marital Status**	Married/de facto	75.5	(75.2, 75.8)	75.0	(74.8, 75.2)
	Widow/divorce/separ.	19.8	(19.5, 20.1)	19.0	(18.8, 19.2)
	Single	4.7	(4.5, 4.9)	6.0	(5.9, 6.1)
**Health**	Private	57.0	(56.6, 57.4)	52.5	(52.3, 52.7)
**Insurance**	DVA or HCC	29.8	(29.4, 30.2)	29.5	(29.3, 29.7)
	None	13.2	(12.9, 13.5)	18.0	(17.8, 18.2)

* all characteristics were significantly associated with use of glucosamine (p<0.0001).

Table 2 shows a comparison between participants who used glucosamine and those who did not use glucosamine by health status characteristics. Use of glucosamine was highest among those participants who never smoked, drank 7–13 alcoholic drinks per week, whose overall health and quality of life were rated as being very good, and who reported being treated for osteoarthritis, osteoporosis, asthma, high blood pressure, high cholesterol, and thyroid problems in the previous months. Conversely, participants who reported being treated for cancer, heart attack or angina, and other heart disease in the previous month were all lower users of glucosamine (p<0.0001 for all but ‘other heart disease’, p = 0.0004).

**Table 2:** Health status characteristics of people aged 45 years and older by glucosamine use.

		Use of Glucosamine			
Health Status Characteristics*		Yes		No	
		(n = 58,630)		(n = 208,214)	
		%	(95% C.I.)	%	(95% C.I.)
**Smoking Status**	Current smoker	3.9	(3.7, 4.1)	8.2	(8.1, 8.3)
	Former smoker	36.4	(36.0, 36.8)	35.5	(35.3, 35.7)
	Never smoked	59.6	(59.2, 60.0)	56.3	(59.4, 59.8)
**Alcohol**	0–6 drinksper week	62.1	(61.7, 62.5)	62.4	(62.2, 62.6)
**Consumption**	7–13 drinksper week	19.9	(19.6, 20.2)	18.6	(18.4, 18.8)
	14–20 drinksper week	11.3	(11.0, 11.6)	11.0	(10.9, 11.1)
	≥21 drinksper week	6.7	(6.5, 6.9)	7.9	(7.8, 8.0)
**Overall Health**	Excellent	14.0	(13.7, 14.3)	15.4	(15.2, 15.6)
	Very Good	38.6	(38.2, 39.0)	36.4	(36.2, 36.6)
	Good	34.8	(34.4, 35.2)	33.5	(33.3, 33.7)
	Fair	11.1	(10.8, 11.4)	12.3	(12.2, 12.4)
	Poor	1.5	(1.4, 1.6)	2.4	(2.3, 2.5)
**Overall Quality**	Excellent	23.7	(23.4, 24.0)	23.7	(23.5, 23.9)
**Of Life**	Very Good	38.8	(38.4, 39.2)	36.9	(36.7, 37.1)
	Good	28.3	(27.9, 28.7)	28.3	(28.1, 28.5)
	Fair	8.0	(7.8, 8.2)	9.2	(9.1, 9.3)
	Poor	1.2	(1.1, 1.3)	1.9	(1.8, 2.0)
**Osteoarthritis**	Yes	16.6	(16.3, 16.9)	5.7	(5.6, 5.8)
	No	83.4	(83.1, 83.7)	94.3	(94.2, 94.4)
**Osteoporosis**	Yes	7.8	(7.6, 8.0)	5.2	(5.1, 5.3)
	No	92.2	(92.0, 92.4)	94.8	(94.7, 94.9)
**Asthma**	Yes	5.3	(5.1, 5.5)	4.6	(4.5, 4.7)
	No	94.7	(94.5, 94.9)	95.4	(95.3, 95.5)
**Cancer**	Yes	2.4	(2.3, 2.5)	2.9	(2.8, 3.0)
	No	97.6	(97.5, 97.7)	97.1	(97.0, 97.2)
**High Blood**	Yes	26.4	(26.0, 26.8)	23.8	(23.6, 24.0)
**Pressure**	No	73.6	(73.2, 74.0)	76.2	(76.0, 76.4)
**High Cholesterol**	Yes	16.6	(16.3, 16.9)	14.8	(14.6, 15.0)
	No	83.4	(83.1, 83.7)	85.2	(85.0, 85.4)
**Heart Attack or**	Yes	2.3	(2.2, 2.4)	2.7	(2.6, 2.8)
**Angina**	No	97.7	(97.6, 97.8)	97.3	(97.2, 97.4)
**Other Heart**	Yes	2.6	(2.5, 2.7)	2.8	(2.7, 2.9)
**Disease**	No	97.4	(97.3, 97.5)	97.2	(97.1, 97.3)
**Thyroid**	Yes	6.0	(5.8, 6.2)	4.7	(4.6, 4.8)
**Problems**	No	94.0	(93.8, 94.2)	95.3	(95.2, 95.4)
**Anxiety**	Yes	4.2	(4.0, 4.4)	4.3	(4.2, 4.4)
	No	95.8	(95.6, 96.0)	95.7	(95.6, 95.8)
**Depression**	Yes	6.6	(6.4, 6.8)	6.9	(6.8, 7.0)
	No	93.4	(93.2, 93.6)	93.1	(93.0, 93.2)

* all characteristics were significantly associated with use of glucosamine (p<0.001), with the exception of anxiety (p = 0.3856) and depression (p = 0.0178).

Table 3 shows the results of the multiple logistic regression modelling. The odds of glucosamine use was 3.31 (95% CI: 3.18, 3.45) times greater for those participants reporting treatment for osteoarthritis compared to those not reporting osteoarthritis. The odds of glucosamine use was 0.85 (95% CI: 0.79, 0.91), 0.79 (95% CI: 0.73, 0.86) and 0.82 (95% CI: 0.76, 0.89) times lower for those participants reporting cancer, heart attack or angina and other heart disease, respectively. Those participants who rated their overall health as being very good (OR = 1.53; 95% CI: 1.36, 1.72) or good (OR = 1.52; 95% CI: 1.36, 1.71) were more likely to use glucosamine as were those participants who rated their overall quality of life as being excellent (OR = 1.28; 95% CI: 1.23, 1.45) or very good (OR = 1.25; 95% CI: 1.10, 1.41).

**Table 3:** Multiple logistic regression model for predicting use of glucosamine in people aged 45 years and older.

Factor		OddsRatio	95% C.I.	p-value		
**Sex**	Male	1.00	–			
	Female	1.39	1.35, 1.43		<0.0001	
**Age**	45–49	1.00	–			
	50–59	1.68	1.61, 1.75		<0.0001	
	60–69	2.34	2.24, 2.45		<0.0001	
	70–79	2.42	2.29, 2.54		<0.0001	
	80+	2.03	1.91, 2.15		<0.0001	
**Place of**	Major city	1.00	–			
**Residence**	Inner regional	1.05	1.02, 1.08		<0.0001	
	Outer regional	1.09	1.05, 1.13		<0.0001	
	Remote/very remote	0.83	0.75,0.92		<0.0001	
**Annual**	< $20000	1.00	–			
**Household**	$20000–$49999	1.27	1.23, 1.32		<0.0001	
**Income**	$50000–$69999	1.28	1.22, 1.34		<0.0001	
	≥ $70000	1.21	1.16, 1.27		0.0546	
**Insurance**	Private	1.00	–			
	DVA or HCC	0.86	0.83, 0.89		0.7908	
	None	0.75	0.72, 0.78		<0.0001	
**Smoking**	Current smoker	1.00	–			
**Status**	Former smoker	1.84	1.74, 1.95		<0.0001	
	Never smoked	1.81	1.70, 1.92		<0.0001	
**Overall**	Poor	1.00	–			
**Health**	Fair	1.36	1.22, 1.52		0.3362	
	Good	1.52	1.36, 1.71		<0.0001	
	Very Good	1.53	1.36, 1.72		<0.0001	
	Excellent	1.34	1.19, 1.51		0.5510	
**Overall** **Quality**	Poor	1.00	–			
**Of Life**	Fair	1.16	1.02,1.31		0.5594	
	Good	1.20	1.06, 1.36		0.1894	
	Very Good	1.25	1.10, 1.41		0.0005	
	Excellent	1.28	1.23, 1.45		<0.0001	
**Osteoarthritis**	No	1.00	–			
	Yes	3.31	3.18, 3.45		<0.0001	
**Cancer**	No	1.00	–			
	Yes	0.85	0.79, 0.91		<0.0001	
**Heart** **Attack or**	No	1.00	–			
**Angina**	Yes	0.79	0.73, 0.86		<0.0001	
**Other Heart**	No	1.00	–			
**Disease**	Yes	0.82	0.76, 0.89		<0.0001	

Participants were more likely to use glucosamine if they were: former smokers (OR = 1.84; 95% CI: 1.74, 1.95) or never smoked (OR = 1.81; 95% CI: 1.70, 1.92), compared to current smokers; had a household annual income of $20000–$49999 (OR = 1.27; 95% CI: 1.23, 1.32) or $50000–$69999 (OR = 1.28; 95% CI: 1.22, 1.34), compared to those with an annual income of <$20000; were aged 60–69 years (OR = 2.34; 95% CI: 2.24, 2.45) or 70–79 years (OR = 2.42; 95% CI: 2.29, 2.54), compared to those aged 45–49 years; and were female (OR = 1.39; 95% CI: 1.35, 1.43). In comparison to those participants who live in a major city, the odds of glucosamine use are greater for those living in inner regional areas (OR = 1.05; 95% CI: 1.02, 1.08) and outer regional areas (OR = 1.09; 95% CI: 1.05, 1.13), but lower for those living in remote or very remote areas (OR = 0.83; 95% CI: 0.75, 0.92). Participants with no health insurance were 0.75 (95% CI: 0.72, 0.78) times less likely use glucosamine compared to participants with private health insurance. The Hosmer-Lemeshow goodness-of-fit test statistic for this model is statistically significant (χ^2^ = 34.5; p<0.0001).

## Discussion

Our research findings show that 22% of the study participants, who are aged 45 years and older, consume glucosamine. This is far higher than the level of use recorded by a 5-year prospective study in Canada (4.7% for women; 8.2% for men) [24]. One possible explanation for this difference is that the Canadian study covered the period between 1996 and 2001 and glucosamine has become far more popular since this time given emerging evidence that suggests potential benefits and the subsequent media coverage [12]. However, the level of glucosamine use identified from our study is similar to that of the United States as identified in a national CAM survey in 2007 [6] as well as findings from three Australian studies reporting glucosamine use ranging from 15–33% [9]–[11] and confirms that glucosamine is one of the most commonly used CAM in Australia.

Our study findings, that being female, of increased age and completing advanced education are all associated with glucosamine use, are also in line with the results of previous glucosamine consumption research [24] as well as the identified predictors of CAM use more broadly [6], [38]–[41]. The association of glucosamine use with higher annual income and private health insurance (optional in Australia) is not too surprising given that glucosamine, like many CAM products, is not subsidised by the Pharmaceutical Benefits Scheme (a Federal government program providing subsidised prescription drugs to residents) and attracts an added 10% goods and services tax in Australia.

The association of glucosamine use with osteoarthritis may reflect the increase in popularity of these products as alternative treatments for this particular condition [13]. Glucosamine is largely promoted and recommended for arthritis, so this finding is not unexpected [12]–[14]. However, the finding of a negative association of glucosamine use with cancer, heart disease and other cardiovascular conditions is revealing. A systematic review of the prevalence of dietary supplement use in cardiac patients found that while 36% of patients reported supplement use (across 20 studies), only 4% of patients (across 8 studies) had reported taking glucosamine/chondroitin [42]. Lower use in cardiac patients may be related to patients’ concerns and/or professional advice regarding the potential for drug interactions with prescription antiplatelets or anticoagulants [43] or simply that glucosamine is not advocated for the treatment of heart disease. This is an issue that warrants further investigation.

Our study also reveals that Australians who have better quality of life/health ratings or a healthy lifestyle (e.g. non-smoking) have greater odds of glucosamine use. Such findings are interesting in as much as they may support the idea that glucosamine is used not only for symptom management but also as a preventive therapy [24]. This is further supported by the fact that Australian health professionals support the use of glucosamine for preventative purposes (20.6% of GPs and 23.4% of community pharmacists surveyed recommended glucosamine for prevention) [7]. Given these results it would be useful for future studies to differentiate between the therapeutic and preventive use of glucosamine and to explore the reasons for patient’s different use of CAM.

Finally, the study results reveal a major geographical difference in the consumption of glucosamine with use more likely among those living in rural areas (though not in very remote areas). This spatial differentiation in the consumption of supplements has not been analysed in previous work due a focus upon a much higher proportion of urban residents compared to rural residents [24]. The finding of higher glucosamine use in rural regions is in line with previous studies examining the spatial determinants of CAM use more broadly [38], [44], [45]. The discovery of an urban-rural difference in glucosamine use suggests that further research is needed to explicate the characteristics and diversity of such use in different geographical settings.

The study finding of a high prevalence of glucosamine use among older Australians suggests the need for policy attention regarding glucosamine consumption and CAM use more broadly. The association of glucosamine use with osteoarthritis suggests that consumers may be critically selective in their CAM consumption, employing glucosamine primarily as indicated rather than extending use of popular supplements to non-indicated conditions. The negative association of glucosamine use with cardiovascular disease may suggest that consumers are taking heed of publicly available information regarding interactions between glucosamine and medicines used in the treatment of cardiovascular disease. Given glucosamine is the most commonly recommended CAM by GPs and community pharmacists in Australia [7], [8], future research is needed on the role healthcare professionals play in the decision-making of patients’ use of CAM, as well as the information sources that patients use to make decisions related to self-prescription.

The high use of glucosamine in Australia also highlights a need for more detailed policy attention on CAM. Differences in the formulation of glucosamine products – either through differing manufacturing processes or different raw or constituent materials – is identified as a factor contributing to the heterogeneity in clinical trial results [23], [24]. Moreover, evidence suggests glucosamine hydrochloride products are associated with more negative results than glucosamine sulphate products [16], [17], [46], [47]. However, Australian regulatory and health authorities have not traditionally made distinctions between specific formulations, and little is known about public preferences between formulations. As such, health care policymakers and researchers need to be alert to the varying quality of supplement products employed by the general public in order to maximize potential benefit and minimize possible harm. As such, glucosamine, along with other complementary medicines, should not be evaluated as a broad category but in terms of specific commercial formulations.

The interpretation of our findings is limited by the fact that health and health care use is self-reported by the participants and as such study results may be open to the effects of recall bias or self-diagnosis. The interpretation of the study findings is also limited by the fact that the variable used from the 45 and Up Study survey was based on those reported as being ‘treated in last month’ rather than ‘ever been diagnosed’. Further, the 45 and Up study questionnaire was not developed for the specific purpose of examining glucosamine use. As our research is a secondary analysis of the 45 and Up data, we are limited to the questions devised by the 45 and Up Study investigators and as such, some potential factors predicting glucosamine use may be missing. Nevertheless, these limitations are outstripped by the insight gained through collecting and analysing such a large sample of adults aged 45 years and older.

### Conclusion

Glucosamine has become a popular alternative treatment for osteoarthritis and this study has estimated that a considerable proportion of the study participants (aged 45 and over) consume glucosamine. The high utilisation of glucosamine may have important clinical ramifications; highlighting the need for primary care providers to discuss self-prescribed CAM use (including glucosamine use) with all their patients, not just those that they suspect of being users. Additionally, given the product variability and quality issues surrounding glucosamine preparations, the high utilisation of glucosamine highlights the need for further attention to these issues to ensure effective application in the treatment or prevention of osteoarthritis.

Given the concerns raised over potential drug interactions between glucosamine and common pharmaceuticals such as warfarin, it is important for the medical profession to be aware of the use of this dietary supplement among their patients and for researchers to further investigate the reasons for and details of glucosamine use.
